# 

**Published:** 1828-04-01

**Authors:** 


### BIBLIOGRAPHICAL RECORD.

[*Jan.* 1*st, to April* 1*st,* 1828.]

1. On the Mortality and Physical Management of Children. By Mr. Robertson.
2. The Good Nurse. Second Edition.
3. Essay on Stricture of the Rectum. By F. Salmon. 8vo.
4. Traité General d'Anatomie Comparée. Par J. F. Meckel. T. Premier.
5. Sir A. Halliday on Lunatics and Lunatic Asylums. 8vo. pp. 100.
6. The Water Question. By J. Wright. 8vo.
7. Memoir on Mutual Instruction. Translated from Beuillac. 12mo. pp. 36.
8. Anatomical Description of the Human Eye. By A. Watson, Esq. 4to.
9. Oratio Harveiana. By Dr. Bree.
10. Dr. Stewart on Tendency to Disease of Body and Mind.
11. On the Preparation of New Remedies, &c. By J. Houlton. 12mo.
12. Supplement to the Pharmacopœia. By S. Gray. 8vo. pp. 528.
13. Memoirs of the Life of John Mason Good. 8vo. pp. 472.
14. Physiological Illustrations of the Organ of Hearing. With Plates.
15. No. IV. of Flora Medica.
16. Researches into the Diseases of India. By J. Annesley, Esq.
17. Manual of the Physiology of Man. Translated from Hutin. 12mo.
18. Thompson's Conspectus. Seventh Edition. Price 5s. 6d.
19. On Derangements of the Digestive Organs. By W. Cooke. 8vo.
20. Observations on Cow-Pox. By Henry Edmonston. 8vo.
21. Practical Observations on Insanity. 8vo.
22. Cases Illustrative of the Effects of Acupuncturation. By J. Morss Churchill. 12mo.
23. An Essay on Diseases of the Jaws. By L. Koecker. 8vo.
24. Dr. Burne on Fever. 8vo. pp. 248. 1828.
25. Mr. Abernethy's Lectures. In one closely printed volume. 8vo. Price 14s.
26. Mr. Warren on the Nature and Properties of Living Animals. 1828.
27. Notes on the Epidemic Cholera of India. By Dr. Kennedy.

### *Additional Subscribers since last Quarter.*

Acret, W. H. Esq. Surg 47, Torrington Square.
Armiger, Mr. J T. Assistant Surgeon to the London Hospital.
Baike, Dr. Madras Establishment.
Barrack, Mr. Surg. Gower Street North.
Beale, Mr. Bedford-street, Covent Garden.
Bishopp, Dr. Thornby, Northamptonshire.
Borton, Dr. Malton, Yorkshire.
Carrick, Mr. Kensington.
Clutterbuck, Mr. C. Surgeon, Gloucester.
Cobb, Mr. Robert.
Dermott, Mr. Lecturer on Anatomy, &c. Great Pulteney Street, Golden Square.
Edye, Mr. John, Surgeon, Exeter.
Gibson, Mr. J. E. Surgeon, Dumfries.
Gooch, Dr. Berners Street, London.
Horwood, Mr. John, Student, Borough.
Huxtable, Mr. W. Surgeon, Hackney.
Inman, Mr. Surgeon, Strand.
Jephson, Mr. (late of Leamington) Glasgow.
Kennedy, Alex. G. M.D. Licentiate of the Royal College of Surgeons, and Member of the Royal Medical Society of Edinburgh, Uttoxeter.
Lambden, Mr. Surgeon, Coningsby, Lincolnshire.
Litchfield, Mr. John Charles, Theatre of Anatomy, Sidmouth Street.
Luke, Dr. Cavendish Square.
M'Kittrick, Mr. John, H.M.S. Wellesley, Mediterranean.
M'Munn, Dr. Assistant Surgeon, 10th Foot, Cork Barracks.
Maule, John, M. D. Surgeon, Wilts, Militia, Marlborough.
Morley, V. W. Esq.
Mullins, Mr. New Street, Dorset Square
Myddleton, Dr. Wells.
Nisbett, Dr. of the University of Edinburgh.
Passingcomb, Mr. J. Surgeon, Bishop Weelsham.
Pattison, Prof. Granville Sharpe, Pall Mall.
Quin, Dr. Marlbro' House.
Roden, Mr. Surgeon, Stafford.
Sankey, Mr. F. H.
Saunder, Mr. 12, North Buildings, Finsbury Circus.
Skegg, Mr. (at Messrs. Jones & Inman's, Strand.)
Stanley, Mr. Lincoln's-Inn Fields.
Struve, Dr. German Spa, Brighton.
Taynton, Mr. Surgeon, Bromley, Kent.
Thomson, Dr. A. T. Thayer Street, Manchester Square.
Tucker, Mr. Surgeon, John St. Adelphi.
Waller, Mr. Surgeon, and Licentiate of the Apothecaries' Company, Kendal.
Williams, Mr. Allan, Surgeon, St. Thomas's Street, Southwark.
Wintle, Mr. W. F. Student, Borough.

### INDEX.

A.

Abdominal contusion, violent case .... 506
Abercrombie, Dr. on diseases of the brain 337
Abernethy, Mr. his lectures.......... 570
Abscess between the bladder and pubes, 451
Acute Bronchitis, at the Val de Grace.. 143
Amesbury, Mr. on non-union of fracture 524
Amputation on the field and in hospital, 461
Amputation, Mr. C. Bell on.......... 518
Andral, M. on peritoneal inflammation, 66
Aneurism, essential distinctions of.... 153
Aneurism, false of the heart; Dr. Elliotson .......................... 174
Aneurism—ligature *ultra aneurisma*.. 467
Angina pectoris, M. Laennec on ..... 428
Annesley, Mr. on the diseases of India, 409
Aortic aneurisms, case of............ 559
Apothecaries' Hall, regulations of the.. 242
Arm and shoulder presentations, Dr. Lee on ........................ 470
Articular inflammation, *Balfourian* practice in........................ 156
Ascites cured by wine vapour........ 508
Asthma, new remedy for............. 220
Asthma, Mr. Abernethy's pathology of, 573
Auscultation, Laennec's treatise on ... 103
Auscultation, debates on ............. 470
Axillary aneurism, case of ........... 556

B.

Barras, Dr. on gastralgia and enteralgia 45
Bell, Mr. on secondary hæmorrhage.... 565
Biliary ducts, diseases of the ......... 529
Bladder, remarkable disease of the .... 492
Bleeding in erysipelas, successful case of, 475
Brain, fatal disease of, mistaken ...... 454
Breschet, M. his cases of disease of the heart .......................... 151
Bright, Dr. his reports of medical cases 90
Bright, Dr. on dropsical effusion...... 313
Brodie, Mr. his case of diseased breast, 126
Brodie, Mr. his case of inguinal aneurism 547
Bronchotomy, Dr. Cullen on ........ 496
Broussais, M. his hospital report...... 142
Broussais, M. Casimir on Gymnastics.. 159
———— M. on English medicine.... 205

C.

Caries, Dr. Nicol on the treatment of.. 441
Catalepsy, Mr. North's case of........ 217
Cerebral hæmorrhage, curious cases of, 148
Cervical vertebræ, luxation of the .... 222
Cervical vertebræ, fracture of........ 452
Cervix femoris, fracture of the ...... 43
Chambers, Dr. on pulmonary abscess.. 138
Chloride of Lime, Mr. Davidson on.... 566
Chloruret of lime, Dr. Reid on........ 38
———————uncertain composition of ............................ 43
Chronic enteritis, the rage in France.. 47
Chronic peritonitis, some well-marked cases of ........................ 71
Chronic affections of the brain, Dr. Elliotson on .................... 448
College of Physicians, celebrated memorial to the ...................... 233
College of Physicians *v.* Dr. Harrison.. 248
College of Physicians *v.* Dr. Harrison.. 479
College of Physicians, steps taken by .. 560
College of Surgeons, its last curriculum, 515
College curriculum, examination of it.. 545
Common iliac artery, ligature of the .. 472
Compound fracture of the thigh—mortification ....................... 533
Compound fracture, Dr. Young's mode of treating ...................... 554
Compression in articular inflammation, 156
Congestive form of yellow fever, sketch of the.......................... 77
Contagion, means of guarding against, 64
Coroner, duties of the .............. 504
Corporation laws, are they favourable to talent?........................ 37
Coup-de-soleil, Mr. Mitchell on ...... 486
Cranial bones, peculiar affection of the, 522
Cranium, peculiar affection of the .... 489
Cullen, Dr. on bronchotomy .......... 496
Cutaneous diseases, Drs. Graves & Stokes on ........................ 465

D.

Delirium tremens, Dr. Coates on...... 457
Delirium tremens, discussion on...... 484
Delirium, treated by mercury ........ 503
Denmark, Mrs. postscript to her case .. 281
Denmark, Mrs. strange statements respecting ....................... 468
Diabetes mellitus, discussion ........ 545
Diagnosis of hernia, discussions on the, 575
Digestion, important experiments on .. 209
Disease of the breast, two cases of .... 126
Diseases of the jaws, Mr. Koecker on.. 425
Disease of the heart, valvular, case of.. 463
Disease of the lungs—remarkable case, 487
Diseases of the brain, &c. Dr. Abercrombie on...................... 337

Disease of the heart, Mr. Maynard's case of .......................... 474
Dislocations of the vertebræ, Mr. Lawrence on.......................... 320
Dissecting-room, pathology in the .... 542
Dorsal vertebræ, fracture and depression of the .......................... 176
Drainage and tillage, their effects on health.......................... 20
Dropsical effusion and liver disease.... 313
Dropsical diseases, pathology of ...... 92
———————— treatment of ...... 101
Duncan, Dr. on empyema, &c........ 131
Duration of pregnancy, tables on the.. 232
Dysentery, cases of, treated by the chloruret of lime .................... 42

E.

Earle, Mr. his case of hernia ........ 170
Eclectic review on peripneumony..... 289
Education, medical, inquiry into it.... 379
Education, importance of to the medical man .......................... 28
Effects of fear, M. Hellis on the ...... 462
Elliotson, Dr. on the sulphate of copper, 403
Emphysema of the heart, curious case of, 223
Empirics, tenderness of the College to.. 34
Empyema and pneumo-thorax, Dr. Duncan on .......................... 131
Empyema, M. Gasc's case of.......... 137
Empyema, operation for, repeated .... 224
Encephalon, M. Raikem on disease of the 178
Encysted dropsy cured, case of....... 505
Endermic medication, M. Martin on.... 158
Endermic medication .............. 203
Enlargement of the upper lip, case of.. 202
Epidemic of Groningen, Prof. Barker on the.......................... 189
Epilepsy, sulphate of copper in ....... 552
Epileptic, dissection of an............. 136
Epiploon, remarkable disease of the.... 184
Erysipelas phlegmonodes, incisions in.. 218
Erysipelas treated by incisions........ 555
Erysipelas treated by punctures ...... 514
Erysipelas, Sir A. Carlisle on ........ 569
Exhalation of blood from the stomach.. 216
Exomphalos, strangulated, case of .... 479
External iliac, ligature of the.......... 557
Extirpation of the Uterus, Dr. Hesse on, 195
Extirpation of the kidneys, results of.. 208
Extra-uterine pregnancy, case of...... 231

F.

Faulkner, Sir A.B. his rambling notes, 27
Febrile miasm, its mode of action on the system .......................... 59
Fellows of the College do not *consult* with surgeons.......................... 33
Femoral aneurism—iliac artery tied ... 557
Fever, *latent* period of .............. 61
Fever, Dr. Marsh on................ 55
Fever, discussions on .............. 567
Fever, pathology of ................ 495
Fœtus in embryo, diseases of the...... 507
Flora medica ...................... 120
Forbes, Dr. his 2d edition of Laennec.. 103
Foreign bodies in bodies natural...... 483
Fracture of the spine—extension employed.......................... 201
Fractures, Baron Larrey on.......... 202
Fracture of a cervical vertebra........ 478
Fractured bones, non-union of ...... 524
Fractured ribs—empyema .......... 465
Fungoid tumours of the diaphragm and lung .......................... 481
Fungoid disease of the breast, case of.. 128
Fungous eruption, curable by mercury, 401
Fungus on the dura mater—hemiplegia, 558

G.

Gambling, effects of ................ 438
Gangrene of the lungs, case of........ 226
Gangrene of the feet, case of ........ 187
Gangrenous erysipelas, odd case ...... 517
Gastralgia, Dr. Barras on............ 45
———— severe case of............ 48
Gastro-enteritis, acute cases of ....... 145
General practitioners vilified by the Lancet .......................... 550
Glottis, fatal case of œdema of the .... 175
Gout, treated by hot-water .......... 221
Guthrie, Mr. his use of the chlorurets.. 184
Gymnastics, observations on their effects 159
———————— description of them as practised here .......................... 162

H.

Haslam, Dr. his lectures ............ 405
Hæmatemesis, discussion on ......... 512
Heart, peculiar case of disease of the .. 151
Hepatic disease, curious case of ...... 560
Hepatic diseases, Drs. Graves & Stokes on 494
Hernia, curious case of, by Mr. Earle.. 170
Hesse, Dr. on extirpation of the uterus, 195
Hip-joint, injury of the .............. 438
———— Mr. Bell on the............ 444
Horner, Dr. on the mucous membrane, 361
Humoral pathology, M. Stultz on the.. 534
Hydatids of the liver, cured by an operation.......................... 459
Hydrarthrosis, Dr. Vilette on ........ 506
Hydrocephalus, Dr. Monro on........ 385
Hydrocele; Baron Larrey's treatment successful .......................... 501
Hydro-pneumo-thorax, case of ....... 462
Hydrophobia, supposed case of ....... 191
Hypochondriasis, cured by ague, case of, 186

I.

Ice to aneurisms .................. 576
Inflammation by contiguity.......... 505
Inflammatory form of yellow fever, Dr. Wilson on .......................... 75
Inguinal aneurism, Mr. Brodie's case .. 547

Injury of the head—trephine applied .. 521
Instrument of justice, sample of the .. 214
Internal strangulation, M. Louis's cases of 129
Intestinal invaginations, M. Buet on .. 154
Intestinum ileum, perforated, case of.. 225
Irish College of Physicians .......... 490

K.

Kidney, state of in dropsical diseases .. 94
——— varieties of diseased structure of the .......................... 99
Koecker, Mr. on diseases of the jaws .. 425

L.

Laceration of the uterus, Mr. Birch on.. 335
Laceration of the urethra—sloughing—death ........................ 453
Laennec, M. on mediate auscultation .. 103
Laennec, review of his chapter on peripneumony ...................... 289
Lancet, falsehoods of the ............ 541
Larrey, B. his treatment of hydrocele.. 501
Laryngitis, M. Martinet on .......... 168
Lawrence, Mr. his letter to Dr. Johnson, 525
Lawrence, Mr. on dislocations of vertebræ ........................ 320
Leech-bites—fatal hæmorrhage from .. 552
Liberality *v.* illiberality.............. 480
Liberty of the press—more law-suits!.. 456
Ligature of arteries, ultra tumorem.... 552
Ligature *ultra* aneurisma, Mr. Lawrence's opinion ........................ 557
Lancet, ratiocination of the.......... 550
Literary justice, specimen of ........ 569
Lithotomy, Panton Square, cases of .. 194
Lunacy—insanity—the speculations of Dr. Haslam .................... 243
Lunatic Asyla, disgraceful scenes in .. 246

M.

Mackintosh, Dr. on Cullen's system ... 273
Maclachlan, Dr. his case of aneurism of the temporal artery ............ 497
Macleod *v.* Wakley ................ 528
Malaria, Dr. Macculloch on.......... 1
———— the cause of various affections, 2
Malaria, Dr. Macculloch on .......... 370
Malingering, Dr. Cheyne on.......... 309
Marshes and swamps, remarks on ..... 11
Marsh, Dr. on fever ................ 55
Medical cases, reports of; by Dr. Bright, 90
Medical botany, Nos. IV, V. VI. of.. 117
Medical education, inquiry into the state of ............................ 379
Medical education, debates on at Guy's, 237
——————— in Edinburgh ...... 239
Medicine and surgery, practice of ..... 434
Medico-chirurgical Transactions, review of ............................ 332
Medico-chirurgical Transactions, review of ............................ 354
Medulla spinalis, softening of the...... 535
Melæna, discussion on .............. 512
Mill-dams, imminent dangers of........ 16
Mind, influence of the stomach on the.. 121
Misrepresentation, disgraceful system of 480
Mis-statements of the Lancet, good specimen .......................... 476
Modus operandi of the yellow fever miasm 87
Monades, Sir. G. Gibbes' speculations on 446
Morbid sensibility of half the body..... 166
Morbid growth in the femur, case of .. 452
Morbid anatomy of the brain, Dr. Monro on ............................ 385
Moxa, sciatica cured by ............ 501
Mucous membrane, inflammation of the 361
Muriate of iron in softening of the stomach ........................ 552

N.

Nævi materni, Messrs. Lawrence and White on ...................... 332
Nævus maternus, Mr. Bennett on ..... 204
Neck of the bladder, inflammation of the 222
Nervous affections of the heart, M. Laennec on .................... 428
Neuroses, M. Barras' theory of the.... 51

O.

Œdematode swelling of the leg, curious case.......................... 450
Ophthalmia porriginosa—Mr. Christian on .............................. 531

P.

Paraplegia spontanea, instance of ..... 192
Paraplegia, Dr. Thomas on............ 447
Paraplegia, Mr. Earle on ............ 354
Paralysis, from contusion of the spine, 203
Paralysis of the left side of the body, case of .......................... 558
Paruria erratica, extraordinary case.... 509
Pathology of the blood, Dr. Clanny on the, 510
Pemphigoid affection of the spleen .... 216
Pericarditis in a child, case of........ 189
Pericranium, peculiar disease of the ... 485
Peripneumony, eclectic review on .... 289
Peripneumony in children, M. Guersent on ............................ 493
Peritonitis, M. Andral on............ 66
———— acute, cases of............ 68
Phlebitis, Dr. Forbes on ............ 230
Phlegmonous erysipelas, Mr. Daniel on, 444
Physic and surgery—fever .......... 571
Pilimiction, cases of ................ 535
Pneumo-gastric nerves, section of the.. 215
Pneumonia, Laennec's treatment of ... 107
Pneumonia, M. Broussais' treatment of, 144
Pneumo-phthisis cyanotica, M. Schonlein on .......................... 193
Popliteal aneurism in both legs, operation 476
Popliteal aneurism—Mr. Brodie's case.. 541
Popliteal aneurism, secondary hæmorrhage .......................... 564

Polypus, malignant, bleeding, case of.. 478
Pregnancy, fatal sickness from........ 149
Prize Hospital Report, Mr. Wickham's, 249
Pulmonary abscess, Dr. Chambers on.. 138
Pulsating tumours of the scalp, cases of, 497
Pupil, state of the, in deep intoxication, 449
Purpura Hæmorrhagica, Mr. Kingsley's case .......................... 207
Purulent depôts, after wounds, &c..... 470
Pylorus, bony tumour of the ........ 508
Pythagoras Redivivus, in the person of Sir G. Gibbs ..................... 213

R.

Rambling notes of Sir A.B. Faulkner.. 27
Rectum, Mr. Salmon on stricture of the 325
Reid, Dr. on the chlorurets of lime.... 38
Researches into the diseases of India, Mr. Annesley's ...................... 409
Rheumatism; Dr. Cazenave on its treatment ......................... 351
Rheumatic inflammation of the thigh.. 502
Rigidity of paralysed members, cause of 183
Rivers, salubrity of the banks of...... 14
Rome, its past and present climate .... 21
Rupture of the uterus, Dr. Smith on .. 359

S.

Sanson, M. on amputation ............ 461
Sarcocele extirpated—tetanus ........ 537
Secondary hæmorrhage, cases of ...... 477
Secondary hæmorrhage, Mr. Bell on.. 564
Sickness, fatal from pregnancy ...... 149
Signs of the times, or epidemics of the day .......................... 227
Soils productive of malaria .......... 7
Spinal disease, Mr. Auchincloss on .... 553
Spinitis, Dr. Potain's case of ........ 185
Stanley, Mr. his prosecution ......... 527
Stephenson and Churchill's Medical Botany ........................ 117
Stethoscope; prejudiced opposers of the 114
Stomach, influence of, upon the mind.. 121
Stomach and bowels, inflammation of its mucous membrane ............... 361
Stomach; its influence on the brain ... 572
Staining of blood-vessels, M. Laennec on 488
Strangulated hernia, Mr. Brodie's cases of 449
——— case of ......... 451
Strangulated hernia, case of .......... 452
Stricture of the œsophagus; hospital of surgery ....................... 169
Stricture of the rectum, Mr. Salmon's essay on ........................ 325
Stricture of the lacrymal duct, Mr. Teale on ........................ 460
Subclavian artery, ligature of the...... 164
Sulphate of copper, Dr. Elliotson on .. 403
Sympathetic apoplexy, remarkable case, 455
Syphilis, Dr. Dzondi's mode of treating, 206

T.

Temporal artery, curious disease of the 499
Tetanus, vapour-bath in ............. 520
——— after extirpation of a sarcocele 537
——— carbonate of potash and opium in 542
Thames water, *purity* of ............ 455
Thoracic diseases—auscultation ...... 481
Thorpe, Mr. his ligature of the subclavian 164
Topography of yellow fever .......... 82
Tracheotomy, new mode of performing 511
Traumatic delirium, M. Dupuytren on .. 435
——— Tetanus, treatment of ...... 439
Tubercles, prize essay on ............ 490
Tubercles, Dr. Kellie on the effects of.. 562
Typhus, Dr. Clanny's views of ...... 510

U.

Ununited fracture, cured by pressure, 555
Urine, chemical properties of in dropsy 102
Urticaria tuberosa intermittens, case of 464

V.

Valerian, extract of in large doses...... 544
Venesection, wondrous virtues of .... 247
Veins of the round ligament, varices of the 543

W.

Wallace, Mr. on a "fungous eruption" 401
Wardrop, Mr. his *last* case of aneurism. . 468
——— his case of disease of the temporal artery ..................... 499
West India fever, Dr. Wilson on ...... 74
Wickham, Mr. his prize hospital report 249
Wound of the knee—amputation ...... 549

Y.

Yellow fever, Dr. Wilson on the cause of 79

